# The Interplay between Tumour Microenvironment Components in Malignant Melanoma

**DOI:** 10.3390/medicina58030365

**Published:** 2022-03-02

**Authors:** Cornelia Amalinei, Adriana Grigoraș, Ludmila Lozneanu, Irina-Draga Căruntu, Simona-Eliza Giușcă, Raluca Anca Balan

**Affiliations:** Department of Morphofunctional Sciences I, “Grigore T. Popa” University of Medicine and Pharmacy, 700115 Iasi, Romania; ludmila.lozneanu@umfiasi.ro (L.L.); irinadragacaruntu@gmail.com (I.-D.C.); simonaelizagiusca@gmail.com (S.-E.G.); raluca.balan@umfiasi.ro (R.A.B.)

**Keywords:** malignant melanoma, tumour microenvironment, extracellular vesicles, microbiota, therapy targets

## Abstract

Malignant melanoma has shown an increasing incidence during the last two decades, exhibiting a large spectrum of locations and clinicopathological characteristics. Although current histopathological, biochemical, immunohistochemical, and molecular methods provide a deep insight into its biological behaviour and outcome, melanoma is still an unpredictable disease, with poor outcome. This review of the literature is aimed at updating the knowledge regarding melanoma’s clinicopathological and molecular hallmarks, including its heterogeneity and plasticity, involving cancer stem cells population. A special focus is given on the interplay between different cellular components and their secretion products in melanoma, considering its contribution to tumour progression, invasion, metastasis, recurrences, and resistance to classical therapy. Furthermore, the influences of the specific tumour microenvironment or “inflammasome”, its association with adipose tissue products, including the release of “extracellular vesicles”, and distinct microbiota are currently studied, considering their influences on diagnosis and prognosis. An insight into melanoma’s particular features may reveal new molecular pathways which may be exploited in order to develop innovative therapeutic approaches or tailored therapy.

## 1. Introduction

Normally located in the basal layer of the epidermis and dermis of the skin, by their ability of melanin synthesis, melanocytes cooperate with neighbouring cells, especially keratinocytes, to protect DNA from ultraviolet light (UV)-induced damage. Although malignant melanoma accounts for about 1% of all skin cancers, its incidence has been constantly increasing in the last two decades, mainly affecting light-skinned persons [1,2]. An estimated 420,000 new melanoma cases per year are registered worldwide [3], representing 5% of all newly diagnosed cancers [4] and exhibiting a large spectrum of locations and clinicopathological characteristics.

Although current histopathological, biochemical, immunohistochemical, and molecular methods provide a deep insight into its biological behaviour and outcome, melanoma is still an unpredictable disease, with poor outcome [5,6,7,8]. Recent progresses in immunomodulatory therapy have been added to the current arsenal in the fight against melanoma, but there are considerable efforts to identify suitable biomarkers for early diagnosis, staging, differential diagnosis, prognosis, and tailored therapy [5,9,10,11].

Microscopic analysis of biopsies or of the surgical specimens is important for the establishment of the histopathological diagnosis and prognosis parameters (tumour thickness or Breslow index, mitotic rate, and ulceration), added to specific immunohistochemical markers, playing together a very important role in melanoma management [5,12].

Additionally, melanoma biomarkers may be classified into different categories, such as diagnostic (showing a higher expression in melanoma cells than in normal tissue), prognostic or predictive markers (showing an increased expression in advanced stages of disease and providing valuable information regarding the treatment response or, on the contrary, correlated to disease recurrence), and progenitor and/or stem cell markers (specific for cell subpopulations that exhibit high carcinogenicity, metastatic potential, and treatment resistance) [5,6,11,13,14,15,16].

Circulating melanoma cells or melanoma-associated extracellular molecules provide noninvasive analytical access, considering the release of proteins and other molecules into the extracellular fluid, and may be considered potential serum biomarkers [5]. Different qualitative and semi-quantitative molecular assays have been used for melanocyte-associated monoclonal antibody (MelanA/MART1), Melanoma-associated antigen recognized by T cells 1 (MART1), and Glycoprotein 100 (gp100) detection [17]. Although debated, blood or serum mRNA levels of tyrosinase, which is involved in melanin synthesis, detected by reverse transcriptase-PCR (RT-PCR), has been significantly correlated with stage progression and the risk of metastatic spread by comparison to other investigated markers [17,18].

Melanoma’s “tumour niche” consists of an ensemble of malignant cells associated with other cells, such as keratinocytes, cancer stem cells, cancer-associated fibroblasts, endothelial cells, and immune cells. This microenvironment has an impact on melanoma cell progression and resistance to therapy [19].

Aggressive melanoma behaviour may be attributed to its heterogeneity, including a population of cancer stem cells (CSCs) [20], currently studied in order to identify their specific markers, which may be further exploited considering their prognostic and therapeutic potential [20].

The dynamic interaction between melanoma cells and adipocytes of the tumour niche may contribute to the establishment of a favourable microenvironment for melanoma growth and progression, especially in obese patients [19]. The therapeutic manipulation of this relationship may offer hopeful perspectives in these patients.

During the last decade, the gut and oral cavity microbiota have been considered a key factor of tumour development by its immunomodulatory function [21]. The unbalanced microbiota may lead to the development of an immune-compromised tumour microenvironment [21]. Current research is aimed at modifying patients’ microbiota as an adjuvant therapy in melanoma [21,22].

Overcoming controversies related to the value of some tumour markers, this review of the literature is aimed to update the knowledge regarding the specific clinicopathological, genetic, and molecular hallmarks, along with providing a comprehensive guide of the tumour microenvironment’s involvement in melanoma prognosis and management.

## 2. Clinicopathological and Molecular Hallmarks Update

### 2.1. General Features

Malignant melanomas derive from an abnormal proliferation of cells originating in melanocytes, cells capable of melanocytic differentiation, and show a characteristic natural aggressive history. Primary melanomas’ presentation is usually associated with a pigmented lesion, but they may also exhibit amelanotic (achromic) features. The most common locations for melanoma are: (i) skin (90%), (ii) mucosa of head and neck (maxillary alveolar ridges, hard palate, tongue, nose, and paranasal sinuses) (8–15%), (iii) gastrointestinal (anorectal region) (<1%), (iv) genital areas (vulvovaginal), (v) meninges and brain (dopaminergic neurons in the substantia nigra and locus coeruleus), and (vi) eye (uvea, conjunctiva, and ciliary body) [23].

Melanoma can affect a wide spectrum of ages belonging to both genders, and it is registering a poor prognosis in patients over 60 years old [24]. Cutaneous melanomas tend to arise in younger people, being related to intermittent high exposure to UV radiation, while mucosal melanomas are more frequent in dark-skinned populations (25–50%) [25,26].

Melanoma has a great metastatic potential, especially the mucosal type, with propensity for lungs, brain, liver, and soft tissues, exhibiting microsatellite, satellite, nodal, or distant metastases pattern and also showing a high ability of local recurrence [25].

### 2.2. Histopathological Characteristics

Although magnetic sequential digital dermoscopy added to clinical examinations are used for diagnosis, the confirmation is based on histological analysis [25]. Melanoma’s presentation may be as melanoma in situ, confined to the epidermis, and infiltrative melanoma, invasive into the dermis. Individuals with multiple atypical nevi (dysplastic nevi) or people with types I and II common nevi, especially with genetic susceptibility or a family history of melanoma, have a high risk for development of melanoma at an earlier age [27].

The most frequent type of melanoma’s growth is the radial growth phase (RGP), which is associated with a better prognosis compared to the other type, the vertical growth phase (VGP). RGP is characteristic for early lesions, being manifested as pigmented plaques or patches expanding horizontally in the epidermis, along the rays of a circle, while VGP is specific for progressive lesions as *bona fide* tumours, which infiltrate the dermis or expand into the epidermis, forming a nodule. Moreover, an early ”tumourigenic” VGP is distinguished by a group of cells within the dermis, wide-reaching to the largest epidermal cell cluster, or a lesion which also shows a proliferation pattern from epidermis to dermis, exhibiting a high mitotic activity [24,25,28].

Current clinicopathological World Health Organization (WHO) classification comprises four major histopathological subtypes: superficial spreading melanoma (SSM) (41%), nodular melanoma (NM) (16%), lentigo maligna melanoma (LMM) (2.7–14%), also known as melanoma arising in a Hutchinson melanotic freckle, and acral lentiginous melanoma (1–5%) [24,29]. The atypical melanocytes may be restricted to the epidermis, showing a lentiginous arrangement at the dermoepidermal junction, or may be restricted to the upper parts of the epidermis (pagetoid or superficial spreading), or can grow along the hair follicles [24,25]. SSM occurs in low cumulative sun damage (CSD), induced by intermittent sun exposure, while its histological diagnosis is made in the presence of pagetoid growth of single cells and nests, exhibiting severe cytological atypia [24,25,30] (Table 1). NM is characterized by an early progression to vertical growth without a radial growth phase and may represent a progression of an acral melanoma or of any other type of melanoma [25]. NM diagnostic criteria include complete loss of maturation, deep mitoses, lymphovascular invasion, and satellitosis, being frequently associated with ulceration [25]. LMM is a high-grade melanoma, with a high-mutation burden and severe solar elastosis which is mandatory for diagnosis, as well as microscopic features consisting of atypical melanocytes with a confluent growth along the dermoepidermal junction, with dermis invasion or with growth along adnexal structures [24,25,31]. Acral lentiginous melanoma can occur in all skin types (palms, soles, and nails), being frequently detected in an advanced stage, while its specific features include: low-mutation burden, epidermal hyperplasia, and characteristic asymmetrical, lentiginous, or nodular pattern of melanocytic cell growth [24,25,32].

A spectrum of lesions has to be considered in melanoma clinicopathological differentials, such as melanocytic nevus (junctional, compound, and dermal), blue nevus, and congenital nevus, along with congenital dermal melanocytosis, a group of lesions represented by Mongolian spot, nevus of Ota, and nevus of Ito [25,33,34,35,36,37]. Sometimes, it is very difficult to differentiate a melanocytic proliferation (genital or oral lentigo) from a non-melanocytic proliferation (basilar epidermal physiological pigmentation) that also displays a strong pigmentation [25]. Additionally, some lesions, such as skin squamous cell carcinoma in situ or Bowen’s disease, early or macular pigmented seborrheic keratosis, actinic keratosis, or junctional dysplastic nevus, are very frequently biopsied, due to strong pigmentation, in order to rule out LMM in situ or invasive melanoma [38]. In some situations, a neurotised melanoma can resemble a neuroid tumour, such as neurofibroma [39]. In our experience, the differentiation of a primary tumour from a metastatic one sometimes raises issues of differential diagnosis, especially when certain organs do not have melanocytes in their structure and their migration from neural crests is not reported.

### 2.3. AJCC Stages and Histological Features in Correlation with Prognosis

Melanoma is stratified according to the American Joint Committee on Cancer (AJCC) staging system (TNM Classification of Malignant Tumors) and WHO classifications, with a direct impact in practice. Thus, both AJCC and WHO systems classify melanoma into five stages (0, IA/B, IIA/B, IIIC, and IV), according to the surgical evaluation of the tumour size and invasion level (0—in situ lesion and stages I–IV), node invasion (stages III–IV), and occurrence of microscopically confirmed distant metastasis (stage IV) [23,24,25,40,41]. In addition to the AJCC staging system, WHO classification includes genetic, genomic, and epidemiologic features of skin melanomas, based on their mutational signatures [25]. Moreover, the molecular data provided by WHO classification result in various histopathologic variants, according to the degree of cumulative solar damage (CSD) of the skin [25].

Traditionally, melanomas were grouped into different subtypes, according to their morphological features, such as spindle, epithelioid, balloon, giant, signet ring, clear, and small cells, along with desmoplastic, rhabdoid, and myxoid [24,25,40,42,43]. Additionally, another distinctive histological feature is the regression of primary melanoma, which may be classified according to the variability of the mononuclear infiltrate, melanophages, and fibrotic process, into the following three categories: early, intermediate, and late stages [44]. However, reported data regarding the tumour–immune system relationship in melanoma regression are controversial. This relationship is particularly intriguing, taking into account that regression has a potential positive impact upon melanoma prognosis, but especially considering that drugs targeting these pathways have shown significant clinical efficacy in multiple tumour types [45]. Recent studies have suggested that the peritumoural inflammatory infiltration can represent a potential therapeutic target, while histological regression stands as an indicator of the immune system’s efficiency in melanoma [46,47].

The diagnosis of melanoma has been traditionally based on Clark’s levels of invasion and Breslow’s index (tumour thickness) that informs on the depth of melanoma invasion. There are five Clark’s levels (level I—confined to the skin surface and epidermis; level II, III, and IV—dermis invasion; level V—subcutaneous fat invasion) and three levels of Breslow’s index (≤1.0 mm—confined to the skin surface and epidermis; >1.0–4.0 mm—dermis invasion; >4.0 mm—subcutaneous fat invasion) [24,25,40,48,49].

AJCC and TNM staging systems and the Union for International Cancer Control (UICC) have improved the accuracy of prognostic prediction scoring, in addition to the presence of histologically recognized ulceration, epidermal defects, mitotic rate (per square millimetre), microscopic satellites, tumour infiltrating lymphocytes (TILs), and lymphatic and perineural invasion, along with tumour regression [24,25,40,50,51]. Moreover, pure RGP melanomas have a very good prognosis, while VGP tumours are prone to metastasis, with metastasis probability correlated to higher stage criteria such as larger thickness, ulceration, microsatellites, increased mitotic rate, lymphovascular invasion, and lack of or minimal TILs [24,25].

### 2.4. Immunohistochemical Markers of Diagnosis

Melanomas display immunostaining characteristics of melanocytic differentiation such as protein melan-A (MelanA) or MART1, microphtalmia-associated transcription factor (MITF), Human Melanoma Black (HMB45), SRY-related HMG-box 10 protein (SOX10), and S-100 protein positivity [24,25,52].

Although most types of malignant melanomas exhibit immunopositivity for these markers, desmoplastic melanomas are negative for MelanA/MART1, MITF, and HMB45. These markers cannot discriminate between nevi and melanomas, and they are not expressed only by melanocytes [25,52,53,54,55] (Table 2).

### 2.5. Genetics and Specific Markers

There are two different molecular pathways leading to melanoma: the first one is associated with sun-exposure, and a second one, an oncogenic pathway, is associated with genetic susceptibility, such as inherited variants of melanocortin-1 or mutations of tumour suppressor genes involved in cellular growth regulation [24,25,56].

Individual genetic susceptibility accounts for about 10% of all malignant melanomas and displays a predisposition pattern with variable gene penetrance (low, medium, and high). The genes that are associated with cases of familial melanoma are: cyclin-dependent kinase inhibitor 2A (*CDKN2A*), melanocortin-1 receptor (*MC1R*), *MITF*, cyclin-dependent kinase 4 (*CDK4*), protection of telomeres 1 (*POT1*), telomerase reverse transcriptase (TERT) promoter region *(TERT)*, adrenocortical dysplasia protein homolog (*ACD*), telomeric repeat-binding factor 2-interacting protein 1 (*TERF2IP*), and BRCA1 associated protein 1 (*BAP1*) [57]. It seems that high penetrance genes (*CDKN2A*, *CDK4*, *POT1*, *TERT*, *ACD*, *TERF2IP*, and *BAP1*) can induce different intrinsic mechanisms, such as cell cycle dysregulation, or even have the capacity to encode two tumour-suppressor proteins, while genes with intermediate penetrance (*MC1R* and *MITF*) act on melanin production and development [57].

MC1R is a receptor for a melanocytic protein involved in skin pigmentation and UV response, being regulated by melanocortin, agouti-signalling protein (ASIP), and β-defensin [24]. Inherited anomalies of MC1R play a significant role in melanoma development [24]. Specific polymorphism of Melanocortin-1 receptor (MC1R) locus, with its variants (R151C, R160W, D294H, R142H, R163Q, and I155T), is potentially mutagenic in melanocytes [57]. Loss of function of this polymorphic gene corresponds to a UV-sensitive, fair skinned, melanoma-susceptible phenotype [58].

Most melanomas are associated with UV exposure, and they are further classified according to the CSD, as low- and high-CSD, added to histopathology assessed by the degree of solar elastosis and different molecular signatures [24,28] (Table 1). Low-CSD melanoma is likely to arise as a result of aberrations of *BRAF* mutation (45%) rather than *RAS* and *NRAS* mutations, which are identified in high-CSD exposure, in 15–30% and 15% of cases, respectively [24,25,56] (Table 3).

Atypical spitzoid tumour and uveal melanoma carry *BAP1* gene mutation and most frequently occur in familial settings [24,25].

By comparison with cutaneous melanoma, mucosal melanoma has a decreased frequency of *BRAF* mutations (<10%) and an increased frequency of mutations of *CD117* or *c-KIT* (40%), along with other somatic mutation (*SF3B1*, *ATRX*, *ARID2*, and *SETD2*). The BRAF-mutated pathway consists of valine replacement with glutamic acid in the *BRAF* gene at location 600 of the polypeptide chain, transforming it in an active kinase, which is responsible for tumour resistance due to reactivation of the mitogen-activated protein kinase (MAPK) pathway [59]. Proto-oncogene *B-Raf* (*BRAF-V600E*) mutation status is required to identify the eligible patients for combined BRAF and mitogen-activated protein kinase (MEK) kinase inhibitors treatment [24].

MAPK/ERK pathway plays an important role in melanoma progression, driving mutations during tumour development via several downstream proteins (RAS, RAF, MEK, and ERK) [60]. Any mutations of this translational or post-translational pathway trigger dissociation of these scaffolding proteins from the MAPK complex leading to cellular proliferation and differentiation of melanomas.

Starting from the assumption that melanoma shares high genetic heterogeneity, currently, detection of melanoma-associated *MAPK* mutations using a next-generation sequencing (NGS) panel is used to construct *MAPK* driver detection gene [61]. Presently, oncogenic alterations in *MAPK* pathway genes in circulating tumour DNA (ctDNA) are used to construct *MAPK* driver detection to check response to therapy or treatment resistance (discussed in detail in Section 7) [62].

RAS proteins are involved in activation of downstream signalling pathways, with significant impact upon cell proliferation, differentiation, and survival, while its variants, with alterations of codon 12, 13, or 61, have an oncogenic effect [63]. *NRAS* represents the most mutated *RAS* isoform, the vast majority of these mutations being found in codon 61, without correlation with UV-damage signature in melanoma [63]. *NRAS* mutations are characteristic for less than 20%of melanoma cases [24].

Considering that the assessment of *NRAS* and *BRAF* mutations is mandatory for the treatment of metastatic melanoma, the specific antibodies against NRASQ61R and BRAF-V600E proteins are immunohistochemically used to provide supplementary data on tumour heterogeneity [64]. Additionally, the theranostic efficiency of combining these two markers in challenging cases of melanoma has been reported [64].

*c-KIT* is a component of RTKs (class III transmembrane receptor tyrosine kinases) [65] and its mutations are rare (less than 3%), being related to acral, mucosal, or chronically sun-exposure melanomas [24]. With four isoforms and being encoded by a proto-oncogene on chromosome 4, position q11–12, human *c-KIT* has most of the mutations located in exon 11 and 13 which lead to MAPK and phosphatidylinositol 3-kinase/serine-threonine kinase (PI3K/AKT) pathways’ induction [65].

Moreover, these mutations do not occur together with *BRAF* or *NRAS* mutations [65].

Immunohistochemically, c-Kit represents a good diagnostic marker for differentiation between benign nevi and malignant melanocytic lesions, as well as between primary and metastatic melanomas [66].

*ATRX* is part of SWI/SNF family of chromatin remodelers, its mutations occurring in different tumours of neural crest cell origin, such as neuroblastoma, low-grade glioma, and glioblastoma, as well as in cutaneous melanoma [67]. *ATRX* alterations or mutations are immunohistochemically expressed as protein loss, with recent studies highlighting the loss of protein immunoexpression during melanoma progression, thus making ATRX a valuable prognostic biomarker [67].

*ARID2 (BAF200)* is also a member of the SWI/SNF chromatin remodeler family, which includes *BAF* (BRG1 or hbrm-associated factor) and polybromo-associated BAF (PBAF) complexes, encoding one PBAF complex subunit [68]. *ARID2* mutations are frequent in melanoma, independent from *BRAF/RAS* mutations status [68]. *ARID2* mutations affect the immune checkpoint inhibitors in melanoma and are linked to an increased infiltration with CD8+ T cells [68].

*SETD2* or *H3* lysine 36 histone methyltransferase is mutated in different human cancers, including mucosal melanoma. It is considered a modulator of different chromatin-regulated processes, such as DNA damage repair and methylation or RNA splicing [69].

Furthermore, some studies have shown that uveal melanoma, similar to cutaneous melanoma, may be associated with variable or incidental sunlight exposure [70,71,72,73]. They tend to be associated with alterations in G-protein-coupled receptors and/or G-α proteins, such as *GNAQ* or *GNA11* activating mutations, followed by CYSLTR2, PLCB4, BAP1, SF3B1, and EIF1AX signalling pathways [23].

Splicing factor 3b subunit 1 (*SF3B1*) represents the largest component of the spliceosome factor 3b (*SF3B*) complex, spliceosome mutations becoming the most fascinating pathway detected in human cancer, including acral and mucosal melanomas, haematological malignancies, and solid tumours, having also prognostic significance [74,75]. *SF3B1* mutations correspond to specific disease phenotypes, considering their involvement in regulation of RNA splicing and in DNA elongation and stability [74]. SF3B1 mutations are characteristics for uveal melanoma and blue nevus-like melanoma, and their assessment has diagnostic and prognostic value [76].

Neurofibromin 1 (*NF1*), a tumour suppressor gene encoding a negative regulator of *RAS*, is the most frequently mutated gene in sun-exposed malignant melanoma, after *BRAF* and *NRAS,* being associated with a high risk of metastasis and a high rate of treatment failure [77]. Discovered in the early 1990s, the somatic *NF1* gene mutations are mainly registered in older male patients and in the desmoplastic melanoma type, being frequently associated with other mutations of the RAS pathway [78,79]. Additionally, a triple wild-type melanoma has been described, being characterized by the lack of *BRAF*, *RAS (N/H/K),* and *NF1* mutations [56]. Recently, Ranzani et al. observed that most *BRAF*/*NRAS* wild-type melanomas are very sensitive to MEK inhibition, regardless of NF1 protein level [80].

In the last decades, other variable genomic alterations have been identified in melanoma initiation and progression, such as the mutation of Ras-related C3 botulinum toxin substrate 1 (*RAC1*), *TERT*, Kirsten rat sarcoma viral oncogene homolog (*KRAS*), Erb-b2 receptor tyrosine kinase 2/4 (*ERBB2/4*), cyclin-dependent kinase inhibitor 2A (*CDKN2A*), tumour protein 53 (*TP53*), and phosphatase and tensin homolog (*PTEN*), along with mitogen-activated protein kinase kinase 1 and 2 (*MAP2K1/2*) [56,81,82,83,84,85] (Table 2).

### 2.6. Putative Melanoma Biomarkers

Considering that circulating melanoma cells may release different proteins or other molecules into the extracellular fluid, these may represent potential serum biomarkers [5]. These biomarkers comprise molecules, which may be pathobiologically considered as enzymes, or soluble proteins and/or antigens, or melanin-related metabolites, or circulating cell-free nucleic acids [5,86,87] (Table 4).

From a pathobiochemical point of view, these biomarkers comprise molecules, including enzymes, such as cyclooxygenase-2 (Cox-2), lactate dehydrogenase (LDH), tyrosinase, matrix metalloproteinases (MMPs), tissue inhibitor of metalloproteinase-1 (TIMP-1), Cathepsin K, CD10, indoleamine-2,3-dioxygenase (IDO), and Legumain [5,86,87]. The other category of melanoma cells’ putative biomarkers is represented by soluble proteins and/or antigens, such as vascular endothelial growth factor (VEGF), vascular endothelial growth factor receptor 3 (VEGFR-3), C-reactive protein (CRP), Galectin-3, Osteopontin, heparin- and chitin-binding lectin YKL-40, melanoma inhibitory activity (MIA), soluble intercellular adhesion molecule 1 (sICAM-1), soluble vascular cell adhesion molecule 1 (sVCAM-1), carcinoembryonic antigen-related cell adhesion molecule 1 (CEACAM), cytoplasmic melanoma-associated antigen (CYT-MAA), melanoma antigen recognized by T-cells 1 (MART 1), melanoma-associated antigen-1 (MAGE), tumour-associated antigen 90 (TA90), S100 proteins, and Sry-related HMG-Box gene (SOX) protein family [5,86,87]. Another type of melanoma biomarkers is represented by melanin-related metabolites, such as L-3,4-dihydroxyphenylalanine (L-DOPA)/L-tyrosine, 6-hydroxy-5-methoxyindole-2-carboxylic acid (6H5MI2C), and 5-S-cysteinyl-DOPA [5,86,87]. The last category of melanoma cells serum markers which has been recently discovered is that of circulating cell-free nucleic acids, such as miRNA-29c and miRNA-221 [5,86,87].

## 3. Inflammatory Microenvironment in Malignant Melanoma

### 3.1. Cancer Inflammasome

The inflammation plays a crucial role in carcinogenesis considering its contribution as a source of cytokines and other tumour growth factors and its ability to eliminate transformed cells [88].

Currently, the inflammatory microenvironment represents a key member in the regulation of different tumourigenesis stages, from early initiation to promotion and distant metastasis [89]. The inflammasome may have favourable roles in the innate immunity or can be abnormally activated in melanoma, as well as in other types of malignancies, with the overexpression of the correspondent effector molecules [89].

The inflammatory tumour microenvironment comprises the population of resident or infiltrating immune cells and inflammatory mediators in the vicinity of malignant cells, nowadays representing a key piece in carcinogenesis, from initiation to promotion and metastasis.

The inflammasomes are proteic complexes consisting of nucleotide oligomerization domain (NOD)-like receptors (NLRs), pro-caspase-1, and apoptosis-associated speck-like protein containing a C-terminal caspase recruitment domain (CARD) domain—ASC, which are crucial for homeostasis maintenance. Inflammasomes are characteristic for different cell types, including antigen-presenting cells and T- and B-lymphocytes, along with cancer cells from the tumour microenvironment, with important roles in carcinogenesis in association with other concurrent factors [90].

Inflammasome stimulation depends on DAMPs/PAMPs’ (danger associated or pathogen associated molecular patterns) perception through cytoplasmic receptors (NLRP1, NLRP3) [91]. Various endogenous host proteins, such as CARD8, or different post-translational and transcriptional mechanisms regulate the inflammasome’s activation [91].

Several studies have demonstrated the influence of single nucleotide polymorphism (SNP) of inflammasome genes in the development and progression of different cancer types [92,93,94]. Although inflammasome constituents are widely expressed in immune and nonimmune cells, their encoding genes’ expression is not always related to the inflammasome formation or activation [95].

The characteristics of inflammasome biology are highlighted by the study of different cells, mouse bone marrow-derived macrophages (BMDMs) being the most investigated cellular type [95,96]. Other cells include mice bone marrow-derived intestinal epithelial cells and neutrophils, human airway epithelial cells, neutrophils, platelets, and peripheral blood mononuclear cells (PBMCs), as well as humans’ and rodents’ CD4+ and CD8+ T cells [95]. Nevertheless, inflammasome type is different among cells and host species. Accordingly, the stimulation of these inflammasomes will induce distinct biological outcomes [95].

The formation and activity of inflammasomes are closely dependent on cell organelles, leading to inflammation and cell death [97]. Several organelles can facilitate inflammasome assembly. Thus, the Golgi complex contributes to NLRP3 enrolment and activation, mitochondria are responsible for NLRP3 recruitment and inflammasome activation, endoplasmic reticulum can promote NLRP3 oligomerization with ASC and NLRP3 signalling regulation [95]. AIM2 and interferon-inducible protein 16 (IFI16) activation are taking place in the nucleus, being followed by their translocation in the cytoplasm, forming a perinuclear inflammasome complex. Opposite to the inflammasome action, which can induce cell death, stress granules are responsible for cell survival and inhibit NLRP3 inflammasome formation [95]. Ribosomes preserve cellular translation and generate inflammasomes and cytoskeleton components, especially the microtubule-organizing centre (MTOC), which contributes to NLRP3 and Pyrin formation, inflammasome translocation, and stability maintenance [95]. This “headquarter” activates numerous apoptotic and inflammatory caspases, cytokine substrates, the plasma membrane rupture protein ninjurin-1 (NINJ1), and the pore-forming protein gasdermin D (GSDMD), followed by inflammation, cellular destruction, and pyroptosis [95,98,99,100].

The innate immune system uses a group of pattern-recognition receptors (PRRs) as inflammasome components, which are expressed in different cell types involved in defence processes, such as dendritic cells, neutrophils, epithelial cells, macrophages, and monocytes [90]. Moreover, PRRs include the nucleotide-binding domain, leucine-rich repeat containing receptors (NLRs), and the absent in melanoma 2 (AIM)-like receptor (ALRs) [101]. The activated inflammasome sensor (NLRP1, NLRP3, NLRP6, NLRP9b, AIM2, caspase-11, or Pyrin) establishes the identity of the inflammasome complex [95]. The protein absent in melanoma 2 (AIM2) is a member of the PYHIN family, with one pyrin (PYD) domain situated at the N-terminus and one or two hematopoietic, interferon inducible, and nuclear (HIN) domains located at the C-terminus. The gene encoding this inflammasome sensor was first identified as a tumour suppressor gene in human melanoma cell lines [102].

ASC recruits pro-caspase-1, which is converted into catalytically active caspase-1 [101] and bioactive subunits p20 and p10 which will generate the bioactive forms of interleukins IL-1β and IL-18 by proteolytic cleavage of pro-IL-1β and pro-IL-18 [101].

Activation of the inflammasomes will subsequently lead to pro-inflammatory cytokine release by adjacent cells and tissues. Persistent inflammation will generate a chronic inflammatory status, which contributes to the pathogenesis of variable diseases, including cancer [103].

### 3.2. Interactions in Melanoma’s Inflammasome

The complex inflammasome’s interactions between different cytokines, endothelial, and tumour cells are contributing to angiogenesis and carcinogenesis, along with invasion and metastasis, in melanoma.

Melanoma involves upregulation of pro-inflammatory cytokines, such as IL-6, IL-8, C-C chemokine ligand 5 (CCL5), and IL-1β [101], along with VEGF [104]. In this regard, melanoma-derived IL-1β works as a stimulator of angiogenesis, tumour growth, invasion, and metastasis [105,106,107,108,109,110,111], the influence of the inflammasomes depending on the tumour cell types [101]. The results of a study performed in a murine experimental model have demonstrated the dual functions of IL-1 and inflammasomes in inflammation-induced skin tumourigenesis [112]. Moreover, norepinephrine (NE) is able to upregulate IL-6, IL-8, and VEGF in C8161 the melanoma cell line, exhibiting an autocrine stimulation along with chemotactic and proangiogenic effects, its value being increased in advanced stage melanomas [104].

Other works have demonstrated that ASC is an inhibitor of carcinogenesis by NF-κB transcriptional activity and IκB kinase α/β phosphorylation suppression in primary melanoma, while up-regulated ASC is stimulating the inflammasome, via IL-1β secretion and NF-κB activity, in metastatic melanoma [113,114]. Moreover, the knockdown of NLR family pyrin domain containing 1 (NLRP1) reduces their tumour-promoter properties, both in vivo and in vitro [113,114,115].

Numerous studies have demonstrated that NLRP3-inflammasome dysregulation can activate the inflammasome-dependent IL-1β expression in human sporadic metastatic melanoma cells [110,116,117]. Furthermore, melanoma tumour growth is linked to the ATP-regulated K+ channel P2 × 7 activity associated with NLRP3-inflammasome stimulation [91,118,119].

Another study has reported that modified gain-of-function variants of inflammasome genes NLRP1 and NLRP3 could increase patients’ risk for developing a sporadic malignant melanoma [94,120]. These findings highlight that the dysregulation of inflammasome activation, with subsequent IL-1β and IL-18 production, is crucial in tumourigenesis, the inflammasome molecules representing potential valuable prognostic melanoma biomarkers [94].

Other studies on SNPs in melanoma genes demonstrated that various cytokines (TNF-a, IL-6, IL-10, IFN-c, and TGF-b1) are involved in melanoma progression and immune escape [121,122]. Some of the cytokines produced by human melanoma cells (IL-6, IL-8, CCL5 (RANTES), CXCL1–3 (MGSA-GROa-c), and monocyte chemotactic protein-1 (MCP-1/CCL2) are associated with tumour invasiveness and aggressiveness [123]. Cytokines activity is stimulated by activated IL-1β [111,124]. Biologically active melanoma-derived IL-1β has a wide range of actions in melanoma tumourigenesis, exhibiting paracrine and autocrine-like activity, increasing IL-1 synthesis in melanoma cells, contributing to macrophages recruitment and to in vitro angiogenesis [110]. It is considered that IL-1β has various effects on different cells of the tumour microenvironment, maintaining survival and proliferation of melanoma cells, immune suppressor cells, and macrophages while promoting invasion and metastasis [125,126,127]. Moreover, IL-1β secretion becomes autonomous as melanoma is progressing [110,111].

Hedgehog (Hh) signalling plays an important role in melanoma pathogenesis, its activity being blocked by wogonin, an active component of flavonoids, in HT144 melanoma cells [128]. It is accepted that wogonin has various inhibitory effects in different melanoma cells, including on invasion and migration of B16F10 cells, melanin synthesis in A375 melanoma cells, or the proliferation and tumour growth of HT144 melanoma cells [129,130]. The anti-inflammatory effect of wogonin in HT144 melanoma is supported by the following activities: (i) pro-inflammatory factors decrease, (ii) anti-inflammatory factors increase, and (iii) inflammatory cytokines expression increase [131]. Additionally, the anti-tumour effects are performed by: (i) glucose consumption decrease; (ii) production of ATP and lactic acid decrease; (iii) kinases’ activities, such as phosphofructokinase (PFK), hexokinase (HK), and pyruvate kinase (PK) inhibition; and (iv) expression of glucose cotransporter-1 (GLUT1), monocarboxylate transporter 1 (MCT-1), and MCT4 inhibition [131].

### 3.3. Melanoma’s Microenvironment Components

The tumour microenvironment (TME) represents a complex biosystem with a great impact on tumour progression, which depends on the spatiotemporal interrelations between malignant and non-malignant cells [132,133], its immune heterogeneity being significant for the prognosis of different types of cancers [134,135].

**Figure 1:**
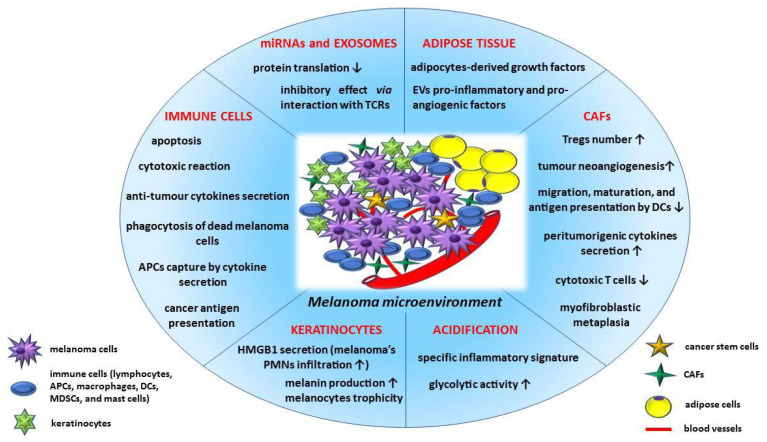
Cellular and extracellular components of melanoma’s microenvironment. The tumour microenvironment (TME) contains numerous cellular and extracellular components, forming together a complex, which supports melanoma development. APCs—antigen-presenting cells; CAFs—cancer-associated fibroblasts; DCs—dendritic cells; EVs—extracellular vesicles; HMGB1- high mobility group box 1 protein; MDSCs—myeloid-derived suppressor cells; miRNAs—microRNAs; PMNs—neutrophils; TCRs—T cells receptors; Tregs—regulatory T cells; ↑—increased level; ↓—decreased level.

The tumour microenvironment (TME) contains numerous immune cells, such as a variable amount of T lymphocytes, as well as B lymphocytes, dendritic cells (DCs), natural killer cells (NK), M1 and M2 type macrophages, mast cells, and myeloid-derived suppressor cells (MDSCs). During the first stages of carcinogenesis, immune cells are involved in apoptosis, anti-tumour cytokines production, and cytotoxic reactions. Thus, NK cells engage antigen-presenting cells (APCs) through cytokine secretion, while DCs, macrophages, and neutrophils are involved in phagocytosis of dead melanoma cells and tumour antigens presentation, activating T cells immune responses [59] (Figure 1).

The activity of the T cells’ main subtypes is mandatory for melanoma remission. Accordingly, the functions of cytotoxic (effector), helper, and regulatory cells are: (i) CD8+ T effector lymphocytes (Teff) recognize antigens via major histocompatibility complex class I (MHC I) molecules, inducing cytotoxicity in melanoma cells and(ii) CD4+ T helper (Th) lymphocytes bind to APCs through MHC II molecules and provide different immune cell types, under the tumour cytokines influence [136,137,138].

CD8+ T cells’ infiltration in metastatic melanoma may be stimulated by the administration of B1 receptor agonist des-Arg9-bradykinin (DABK), considering the involvement of stromal bradykinin signalling and melanoma cells bradykinin receptors in the tumour microenvironment [139,140].

Melanoma cells have great plasticity, allowing the immune escape due to reduced antigen expression, MHC molecules’ decreased level, as well as aberrations in their processing system [136]. As T cells express programmed cell death protein (PD-1) checkpoint receptor, tumour cells are blocking T cell activity via increased production of ligand of PD-1 receptor (PD-L1), leading to an interaction between PD-1 and PD-L1 to produce apoptosis of TILs, stimulating the differentiation of CD4+ into regulatory T cells (Tregs), and inhibiting the immune system response for self-tolerance maintenance [141,142,143]. It has been demonstrated that tumour Treg lymphocytes are correlated with melanoma growth and progression, their recruitment being performed by cancer cells through IL-10, IL-35, and tumour growth factor β (TGF-β) production in order to escape immunity [59,143]. Additionally, it has been shown that β3-adrenergic receptors expressed in melanoma microenvironment mediate Tregs’ and myeloid-derived suppressor cells’ activity, being involved in immune tolerance, in experimental models [144].

Different studies have provided controversial results regarding the activity of B-lymphocytes in melanoma TME. Accordingly, some authors consider that a high density in B cells is characteristic for non-metastatic melanoma, being associated with a better prognosis, while others found that melanoma cells produce fibroblast growth factor-2 (FGF-2) which stimulates B cells to produce insulin-like growth factor-1 (IGF-1), exhibiting a potential resistance to BRAF and MEK inhibitors [145,146]. Another study has emphasized that circulating B lymphocytes produce tumour necrosis factor α (TNF-α) and/or IL-6, these being associated with tumour unresponsiveness and poor survival of melanoma patients who underwent anti-cytotoxic T-lymphocyte associated protein 4 (CTLA4) antibody therapy [147]. The negative correlation between TNF-α expression and immune checkpoint blockade response suggests the role of B cells in tumour growth via inflammatory cytokines production [147].

Tumour-associated macrophages (TAMs), M1 and M2 types, can be important prognostic markers due to their role in tumour cell migration, angiogenesis, and extracellular matrix degradation. M1 macrophages are found in low number in intratumoural infiltrate and have anti-tumoural effects, being activated by Th1 cells and pro-inflammatory factors (granulocyte-macrophage colony stimulating factor—GM-CSF, lipopolysaccharides, and IFN-γ) [148]. M2 macrophages are involved in tumour progression and invasion, as they are mainly identified in early inflammatory infiltrate, being stimulated by Th2 cells and anti-inflammatory stimuli (IL-4, IL-10, IL-13, or monocyte colony-stimulating factor—M-CSF) [149]. Moreover, M2 macrophages can downregulate M1-mediated functions [150].

TAMs possess β3-adrenergic receptors and inducible nitric oxide synthase (NOS2, iNOS) and, as a consequence, their activity may be modulated by NE and nitric oxide (NO), contributing to an increased tumour cells’ growth and invasion [151].

CSCs are related to TME and may recruit TAMs for tumour growth [152]. In this regard, the involvement of CD34-melanoma tumour initiating cells (TICs) in chemoresistance and cancer progression promotion has been demonstrated, through M2 macrophages interaction, along with TGF-β and arginase pathway [152].

Moreover, TAMs may produce adrenomedullin, a vasodilator and stimulator of angiogenesis, a factor involved in TAMs polarization toward M2 type and in melanoma progression [59].

The expansion and migration of MDSCs, which are the precursors of macrophages, granulocytes, and DCs, are influenced by C-C chemokine receptor type 5 (CCR5) ligands (CCL3, CCL4, and CCL5) in melanoma [152]. CD141 DCs from the melanoma immune microenvironment are activating CD8+ T lymphocytes, by CCR7 receptor involvement [153]. Numerous evidence supports that the loss of CCR7 receptor promotes tumour growth, while its increased level is correlated with a better outcome [153]. Furthermore, a reduced DCs number is associated to metastatic melanoma, while their increased amount is suggestive for lack of metastases or low recurrence risk [153].

Neutrophils of the tumour inflammatory infiltrate increase during melanoma progression, their accumulation depending on CXCL1, CXCL2, CXCL3, CXCL5, and CXCL8 molecules, which are stimulated by UV radiations [154]. In an analogous manner to macrophages, neutrophils have also two subtypes: N1, which represent the dominant type of the early melanoma microenvironment, exhibiting anti-tumour activity, and N2 type, occurring in the advanced stages, with immunosuppressive effects [154].

The immune escape of melanoma cells is also produced through reduction in the expression of the main NK receptors (NKp30, NKp44, and NKG2D), which damage the mediated cytolytic anti-cancer activity of NK cells [155].

Different extracellular elements or other cells of the tumour niche may also contribute to the specific TME immune response, such as: fibroblasts, miRNAs or exosomes, acidification, keratinocytes, and adipose tissue (discussed in detail in Section 5.) (Figure 1).

Peritumoural fibroblasts can be converted into cancer associated fibroblasts (CAFs), exhibiting analogous properties to myofibroblasts [156]. During melanoma progression, CAFs may represent an important amount within the tumour cells’ population, displaying variable functions such as immunosuppression, due to TGF-β activity, which include inhibition of migration, maturation, and antigen presentation by DCs, increase in Tregs number, and reduction in the expression of perforin, granzymes, Fas ligand, and IFN-γ in cytotoxic T cells [156]. Fibroblasts seem to be recruited by tumour cells under β3-adrenergic stimulation by NE [151]. Moreover, NE stimulates fibroblasts metaplasia into myofibroblasts, which provide increased tumour cells motility and increased neoangiogenesis [157] along with the release of protumourigenic cytokines, such as FGF-2, IL-6, IL-8, and VEGF [157,158]. CAFs are involved in melanoma progression, metastasis, and drug resistance as a consequence of cell–cell interaction and secretion of extracellular matrix components, growth factors, and cytokines [133,159].

Mast cells are involved in the development of melanoma, considering their ability to react to substance P neurogenic inflammation [160,161]. Consequently, they release different cytokines, proteases, growth factors, biological amines, such as histamine, which reduces the antitumoural defence mechanisms, chemokines, neuropeptides, variable enzymes, and angiogenic factors, such as heparin, VEGF, TGF-β, and IL-8, the latter being demonstrated as a growth factor in different melanoma cell lines [160]. Moreover, their products seem to increase the immunosuppression resulting from UV-B exposure, using a complex mechanism of mast cells stimulation to release their products involving calcitonin gene-related peptide (CGRP), substance P (SP), and keratinocyte-produced nerve factor [161].

MiRNAs, small, non-coding RNAs involved in protein translation attenuation or inhibition, can regulate the melanoma immune microenvironment [162]. Melanoma cells secrete exosomes, which also provide membrane-bound ligands such as PD-L1, with an inhibitory effect of the anti-tumour response via interaction with the T cells receptors [162].

Exosomes, a subtype of extracellular vesicles (EVs), are involved in tumour microenvironment activity and carcinogenesis, mediating the interrelation between cancer cells and CAFs [133]. Several studies highlight the capacity of normal fibroblasts, CAFs, and cancer cells to secrete miRNA exosomes, providing a characteristic intercellular communication within the TME [163,164].

Another extracellular factor which mediates the immune response to cancer cells is acidification, the characteristic lower melanoma pH (6.0–7.0) providing an enhanced glycolytic activity and a specific inflammatory signature [165]. Most of the published data have revealed the immunosuppressive role of acidosis, which develops a “migratory” phenotype of melanoma cells [166,167,168]. The lower melanoma pH is responsible for decreased cytolytic activity of CD8+ T cells and increased secretion of IL-1β by monocytes and TAMs [167], as well as a functional orientation of TAMs toward the M2 type.

The adipose tissue β3-adrenergic receptor also influences the anti-tumour response of immune cells, its upregulation in the melanoma microenvironment resulting in tumour growth stimulation [169].

Keratinocytes can also contribute to the immune escape, influencing the melanoma’s immunosuppressive environment. Usually, the UV-absorbing melanin from keratinocytes protects against melanocytes mutations induced by prolonged radiation exposure, although the UV radiations can also stimulate cancer progression through a different pathway [59]. Recent data have demonstrated that keratinocytes secrete a high mobility group box 1 (HMGB1) protein, which promotes neutrophils infiltration into the melanoma microenvironment, being responsible for melanoma plasticity [59].

Recent data have demonstrated a correlation between keratinocytes and corticotropin-releasing hormone-proopiomelanocortin (CRH-POMC) axis in about 80% of melanomas, along with adrenocorticotropic hormone (ACTH) production in about 70% of melanomas, with α-melanocyte-stimulating hormone (α-MSH) release in over 50% of melanomas [170] and with the functional cell-specific MSH receptor or melanocortin 1 receptor (MC1R) [171]. CRH stimulates melanoma cells invasion via ERK1/2 signalling pathway [172]. Supplementary, desmoglein 1 is involved in the signalling between melanocytes and keratinocytes, with cytokines and POMC production, leading to a high level of melanin and pagetoid melanoma cells spread [173]. However, POMC overexpression reduces the melanoma growth by apoptosis and autophagy via complex α-MSH-HIF-1 α/BCL2 and adenovirus E1B 19-kDa-interacting protein 3 (BNIP3) signalling pathways [174].

Keratinocytes are stimulated by CGRP resulting in stimulation of melanin production and melanocytes trophicity [175]. However, CGRP may induce melanocyte apoptosis via increased Bax/Bcl-2 ratio, while substance P (SP) association with CGRP is inhibiting the process of melanogenesis [175].

Keratinocytes are expressing enkephalins, or opioid receptors (ORs), belonging to G protein-coupled receptors [176,177], while low levels of enkephalin and proenkephalin (PENK) have been detected in melanomas [178]. Moreover, methionine (met)-enkephalin (MENK) has shown melanoma growth inhibition via apoptosis associated with opioid growth factor receptors (OGFrs) increased expression in animal models [179].

Furthermore, it seems that the addictive mechanism of UV exposure is mediated by β-endorphin production in keratinocytes, added to its role in tumour cell proliferation and immune reactions inhibition, by decreasing the amount of tumour lymphocytes [180].

The inflammatory phenotype of the uveal melanoma, the most common primary ocular cancer in adults, is associated with a poor outcome, correlated to a greater number of inflammatory cells populating mainly epithelioid-cell-type tumours, characterized by chromosome 3 loss [181], while the main population in its inflammatory milieu is represented by CD8+ T cells and macrophages [182].

Among the four molecular subsets of uveal melanoma (A, B, C, D), identified in recent studies according to their immunological features and gene expression profiles [183,184], only the subset D shows a characteristic inflammatory phenotype, with excessive infiltration of lymphocytes and macrophages [183,185,186]. Moreover, variant D has an increased metastatic potential and several specific genetic aberrations (monosomy 3, chromosome 8q gain, and BAP1 loss), which seem to be correlated with its specific inflammatory phenotype [186,187]. Recent data regarding the specific immunological and genetic profile of uveal melanoma TME provide a gene-based prognostic signature, with possible impact on prognosis and on targeted therapy perspectives related to metastasis prevention [182].

Another interesting study has assessed the crosstalk between cultured uveal melanoma cells and hepatic stellate cells, demonstrating that metastatic melanoma cells are more sensitive to the paracrine signalling of stellate cells than their non-metastatic category, this interrelation involving profibrogenic interleukins [188]. Thus, metastatic melanoma cells are able to regulate hepatic stellate cells activity, promoting their growth and survival [188].

Tumour progression needs an early and persistent inflammatory response, as cancer cells can modulate the functions of the surrounding cells to favour their growth, invasion, metastasis, and survival [186].

## 4. Melanoma CSCs—The Origin of Heterogeneity, Plasticity, Aggressiveness, and Therapy Resistance

Aggressive melanoma is characterized by variable subpopulations, having multiple phenotype-specific genes and different protein markers. Thus, multiple cellular phenotypes have been identified in aggressive melanoma, such as stem cells, endothelial-like cells, and epithelial-like cells, suggesting a high plasticity [20].

Melanoma CSCs have self-renewal, indefinite proliferation capacities, high tumourigenicity, embryonic-like characteristics, ability to differentiation, and are involved in angiogenesis along with epithelial-mesenchymal transition (EMT) and metastasis [189,190].

The genes involved in vasculogenesis are upregulated in aggressive melanoma, such as EPHA2, CD144, and LAMC2 [189], along with EGFR-Akt-Smad signalling leading to angiogenesis via ID3 regulated cytokine induction [191].

Moreover, the endothelial cell markers seem to be responsible for melanoma cells’ capacity to form de novo vasculogenic-like networks in cultures, with tumour cells situated exterior to the vessels’ basement membrane, being named vascular or vasculogenic mimicry [20]. These channels containing blood, lined by melanoma cells, provide growth advantage and serves as an escape route for malignant cells [7]. Due to the poor expression of integrin α5-subunit, serving as an endostatin target, a failure of angiogenesis inhibitors efficiency has been observed in aggressive melanoma [20].

Melanoma CSCs are associated with a spectrum of markers and molecular pathways which provide them different advantages [5,190,192,193,194,195,196,197,198,199,200,201,202,203,204,205], though they may represent targets for the development of new therapies (Table 5).

Within the notion that malignancies may recapitulate morphogenesis events, Nodal embryonic signalling pathway, a member of TGF-β family, has been identified in aggressive melanoma cells, providing exacerbated tumourigenicity and metastasis capacity [20].

Furthermore, melanoma CSCs are involved in tumour microenvironment modulation by their expression of different miRNAs, along with their capacities of immune escape mechanisms and recurrences due to their limited response to conventional chemotherapy or radiotherapy [20,206,207,208,209,210].

ABC transporters family, and mainly ABCB5, have been shown to induce the tumour progression and multidrug resistance of melanoma cells through regulation of the drug’s efflux in melanoma cells, especially when they are co-expressed with CD133 [211]. The ABC transporters family tumourigenic potential is also supported by C-X-C motif chemokine receptor 6 (CXCR6) expression, another melanoma CSCs marker, particularly associated with asymmetric self-renewal [212]. In the same direction of study, the research conducted by Frank et al. revealed an overexpression of pro-angiogenic factors such as VEGFR1 and VEGF on ABCB5+ and CD133+ melanoma CSCs that promote tumour angiogenesis and support melanoma metastasis [190].

Furthermore, in a study conducted on CD133 + transgenic mice and human melanoma cells, promotion of neovascularisation in tumour microenvironment was induced by the Sox10 high expression, along with that of organic cation transporter (OCT) 3/4 and Nanog homeobox (Nanog) [213].

Sox10, a nuclear transcription factor involved in neural crest cells differentiation into melanocytes and mediation of their malignant transformation, has been observed to be one of the most important melanoma CSCs marker, being expressed in up to 100% of sentinel lymph node micrometastases, comparative to other melanoma cells markers, such as S100 and HMB45 [211].

CD44 is a mesenchymal marker, correlated to EMT, being a receptor which promotes the binding to the extracellular matrix via integrin and, thus, invasion and metastasis processes [214]. CD44 is correlated with insulin-like growth factor-1 (IGF-1), ZEB1, CD29, N-cadherin, and CD105 expression [214].

Aldehyde dehydrogenase (ALDH) enzymes show a common expression in stem cells, including melanoma CSCs, mediating expansion, self-protection, and differentiation [215,216,217]. However, ALDH activity detected in xenografted human metastatic melanoma showed a great variability in expression and, consequently, it is a controversial marker [218]. Additionally, stem-like tumour endothelial cells in human melanoma xenografts expressing strong ALDH show angiogenic capacities [219].

Human ALDH1A family comprises ALDH1A1, strongly expressed in xenografted melanoma, ALDH1A2, and ALDH1A3, strongly expressed in 1205L and A375 human melanoma cell lines and weakly expressed in xenografted melanoma. However, immunohistochemistry and real-time quantitative reverse transcription-PCR (qRT-PCR) reveal that both ALDH1A1 and ALDH1A3 may be expressed in some melanomas [217].

ALDH activity is an important factor in chemotherapy resistance in melanoma and the multi-drug resistance acquisition is correlated to a transition to a drug-tolerant population of cells which strongly express ALDH, along with ABC proteins [220]. As a consequence, a combination therapy using ALDH inhibitor and chemotherapy is increasing CSCs response [218,221]. Furthermore, ALDH may be used as a marker of therapy efficiency or may be selectively targeted in therapy [222,223,224,225,226].

Another application of ALDH expression in melanoma CSCs is the development of a dendritic cells (DCs) vaccine (CSC-DC vaccines) which stimulates tumour infiltration with T cells, along with their products (INF-γ and IL-4) [227,228,229].

Considering that melanoma CSCs also express PD-1 and PD-L1 and CTLA-4, in association with ALDH, consequently, anti-PD-L1 and/or anti-CTLA-4 combined with CSC-DC vaccine showed improved response [230].

Additionally, due to gamma-secretase and B-cell lymphoma 2 (Bcl-2) expression of melanoma CSCs, correlated with that of ALDH, the administration of their inhibitors, i.e., gamma-secretase inhibitors (GSI) and myeloid cell leukaemia sequence 1 (MCL-1)—an inhibitor of Bcl-2—is targeting CSCs [231,232].

The expression of CD20 and CD133 may be induced by NE, which is able to induce stem features in melanoma cells [233].

Melanoma CSCs exhibit a weak immunogenicity and, in addition, show an immunosuppressive effect in the host organism [189].

## 5. Melanoma Cells-Adipocytes “Dialogue”

### 5.1. Hypodermis Role in Cancer Microenvironment

An important component of the cancer microenvironment involved in the progression of melanoma is the adipose tissue, comprising the hypodermis [19]. This area of fat tissue is mainly composed of white adipocytes, associated with other types of cells such as endothelial cells, pericytes, monocytes, macrophages, and stem cells [234]. Current knowledge allows us to consider adipose tissue not only as a lipid storage area but also an inflammatory and endocrine organ [4]. Thus, adipose cells are the source of growth factors such as fibroblast growth factor-21 (FGF-21), hepatocyte growth factor (HGF), IGF-1, VEGF, and endocan, along with insulin-like growth factor-binding protein (IGFBP), leptin, retinol-binding protein 4 (RBP-4), resistin, leukaemia inhibitory factor, IL-6, IL-11, TNF-α, plasminogen activator inhibitor-1 (PAI-1), and TIMP-1 [235,236]. Melanoma cells express surface membrane receptors for these adipocytes-derived factors which support the tumour cells’ proliferation, metastasis, and drug resistance via MAPK, PI3K/AKT, and JAK/STAT pathways [237].

Recent reports show a “bidirectional communication” between melanoma cells and adipocytes, especially in obese patients, consisting of the secretion of high amounts of pro-inflammatory factors such as IL-6, IL-11, TNF-α, monocyte chemoattractant protein (MCP)-1/CCL2, and PAI-1 by the excessive fat [238,239]. Furthermore, the inflammatory profile of subcutaneous adipose tissue is associated with an increased release of leptin and resistin, supporting the progression of tumour cells and increasing the risk of lymph node metastasis [240]. Studies carried out on experimental models support this last finding, showing an increased tumour weight and size as a result of leptin injection into melanoma cells, which induces the activation of AKT-based signal transduction pathway and modulates the activity of fatty acid synthase (FASN), an enzyme involved in de novo synthesis of fatty acids (FA) [237,241]. In addition, adiponectin, with characteristic low levels in obese patients, has the opposite effect, analogous to leptin and resistin in melanoma [242]. Adiponectin induces apoptosis and inhibits cancer cells growth by activation of the AMP-activated protein kinase (AMPK) signalling pathway [242].

These data are also supported by other studies on murine melanoma models which demonstrate a positive correlation between melanoma progression and obesity [237,243,244]. In this respect, excessive subcutaneous white adipose tissue, together with enhanced secretion of pro-inflammatory factors, lead to the progression of melanoma by supporting tumour neoangiogenesis, following the release of pro-angiogenic factors, such as endocan, HGF, and VEGF, added to an altered energy metabolism [4,235]. In addition, other experimental studies revealed that adipocytes co-cultured with melanoma cells induce the secretion of chemoattractant factors (CXCL1, CXCL2, and CXCL5) added to a local immune cell recruitment, especially of M2 macrophages, in the “tumour niche” [236,245,246]. Analogous results have been reported by another research team, leading to the conclusion that skin adipocytes are involved in melanoma cell immune escape, by high expression of PD-L1, which interact with PD-1 molecule on the T lymphocytes membrane [247].

Remarkable results regarding melanoma development have been obtained using mice fed with a high-fat diet [243]. According to the results reported by Pandey et al., a rapid progression of tumour cells is associated with high FASN activity, an increased expression of caveolin-1 (Cav-1), and stimulation of phospho-Akt (pAkt), a protein kinase that plays a critical role in survival and apoptosis regulation [243]. Similar results have been reported by Malvi et al., which demonstrated that the caloric intake restriction and the administration of orlistat, a FASN inhibitor, induces a slowdown of melanoma tumour growth by reducing FASN, pAkt levels, and Cav-1 expression [237]. Furthermore, Cav-1, a membrane-associated protein stabilized by FASN by palmitoylation, acts as a tumour-progressing factor, being involved in cancer-drug resistance along with P-glycoprotein (P-gp), a protein that pumps out drugs from targeted cells [4,237,243]. Moreover, an increase in circulating exosomes expressing Cav-1 has been identified in the serum of melanoma patients, suggesting that Cav-1 may represent a prognostic biomarker [248]. These findings, added to the observation that melanoma cells exposed to adipocyte factors show a reduction in apoptosis induced by cisplatin and docetaxel, mediated by a MEK/ERK and a PI3K/AKT pathway signalling, led to a focus of research on adipocytes involvement in oncologic treatments [249,250].

Recent findings highlight a “metabolic dialogue” in the tumour microenvironment between melanoma cells and adipocytes, based on the observation that the latter provide a local supply of FA, which are transferred to melanoma cells through the fatty acid transport protein 1 (FATP1)/sulute carrier family 27 (SLC27A) family of lipid transporters [233]. These are the energy substrates that support the proliferation of tumour cells, considering their demonstrated role in stimulation of adipocyte lipolysis, leading to cancer-associated cachexia [233]. This latter feature has been demonstrated by morphological studies, which have revealed that adipocytes adjacent to melanoma cells are diminished in size compared to those located far from the tumour [233,251]. Additionally, Zoico et al. has reported adipocytes’ reduction in amount and size, along with that of their lipid droplet content, after few days of melanoma cells and 3T3-L1 adipocytes co-culture [252].

### 5.2. Extracellular Vesicles of Tumour Niche

The “tumour niche” intercellular communication carried out by extracellular vesicles (EVs) which are released by tumour cells and adipocytes has been the aim of recent studies [253,254]. Melanoma cells secrete EVs, which induce cancer progression by downregulation of skin adipocytes activity. These tumour EVs contain miRNAs, including miR-214-3p, which support the formation of a more favourable microenvironment by upregulation of lipogenesis genes, such as fatty acid-binding protein 4 (FABP4), adiponectin, and peroxisome proliferator-activated receptor ɣ (PPARɣ) [251,255]. Additionally, adipocytes are able to differentiate to a fibroblast-like phenotype through Wnt/β-catenin pathway activation and high expression of fibroblast specific markers, such as collagen and *α*-smooth muscle actin (α-SMA) [251,255]. Moreover, melanoma-derived EVs induce EMT and tumour progression through let-7i family miR paracrine or autocrine signalling [256]. In the same direction, tumour-derived EVs have been shown to be involved in the induction of apoptosis of cytotoxic T-cells, induction of M2 polarization of macrophages, and inhibition of cytotoxicity of NK cells in tumour microenvironment [257].

**Figure 2:**
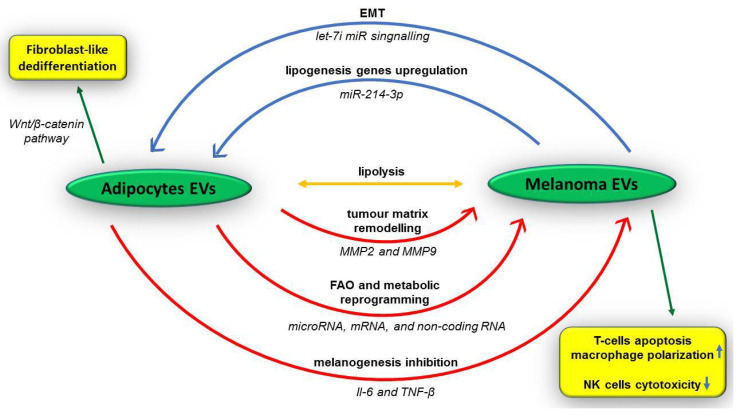
Adipocytes and melanoma cells EVs ’’dialogue’’ in tumour microenvironment. Adipocytes and melanoma cells secrete EVs, which promote tumour progression and metastasis by tumour matrix remodelling, melanogenesis inhibition, fatty acid oxidation (FAO), and metabolic reprogramming into melanoma cells, along with epithelial-mesenchymal transition (EMT) induction, lipolysis, and lipogenesis genes upregulation in fat cells. EVs—extracellular vesicles; EMT—epithelial-mesenchymal transition; FAO—fatty acid oxidation; IL-6—interleukin 6; miRNA—microRNA; MMP—matrix metalloproteinase; NK cells—natural killer cells; T-cells—T lymphocytes; TNF-β—tumour necrosis factor β; ↑—increased level; ↓—decreased level.

All these melanoma-derived EVs actions are supported by the “activity” of skin adipocytes. Thus, adipocytes also release EVs which are internalized by melanoma cells. They contain nucleic acids (miRNA, mRNA, and other non-coding RNAs), lipids, and proteins involved in fatty acid oxidation (FAO), which promote tumour progression through a metabolic reprogramming [258] (Figure 2). In this regard, Lazar et al. has noted that melanoma cells lines SKMEL28 exposed to adipocyte-derived EVs become elongated and develop actin-rich membrane’s protrusions, in a study conducted on a murine model [259].

All these morphological features are associated with adjustment of the mitochondrial network in melanoma cells, with mitochondrial fission and their redistribution to the cell extremities, characteristics which provide a high tumour-progression activity [260,261]. Furthermore, according to the data presented by Clement et al., an increased adipocytic lipolysis is achieved not only in the melanoma invasion front, in locations with a large number of adipocytes, but also by naive adipocytes, which release EVs, which provide both the energy substrate (FA) and the enzymatic equipment (protein) for FAO [258].

Similar results have been reported by another research team, who noticed that B16BL6 mouse melanoma cells exposed to adipocyte-derived factors are associated with higher invasiveness of tumour-cells, as a result of an increased expression of IL-6 and EMT-associated genes, such as MMP9, Snai1, Twist, and vimentin [262]. Additionally, Il-6 and tumour necrosis factor β (TNF-β) synthesized by skin adipocytes induce a paracrine differentiation of melanoma cells, expressed by reduced melanogenesis [235] and promotion of cultured melanoma cells proliferation, by the repression of miR-211 expression [263].

Last but not least, in obese patients, adipocytes participate in “tumour niche” establishment by synthesis of MMPs, especially MMP2 and MMP9, that mediate the remodelling of tumour extracellular matrix, thus promoting motility and invasion of melanoma cells [264]. Besides this, the melanoma cells–adipocytes communication in tumour niche is expressed by an elevated expression of cyclin D1 and Cox-2 oncogenic proteins in tumour cells, along with cell regulatory proteins, such as inhibitor of apoptosis protein-2 (IAP-2), myeloid cell leukaemia 1 (Mcl-1), B-cell lymphoma 2 (Bcl-2), and B-cell lymphoma-extra large (Bcl-xL) [264].

Considering these accumulated data, the dynamic interaction between melanoma cells and adipocytes in tumour niche is still far from elucidation. An important part of this intercellular “dialogue” is performed by adipocyte-derived EVs in association with growth factors, cytokines, and chemokines, which contribute to the establishment of a favourable microenvironment for melanoma growth and progression, especially in obese patients.

The deciphering of the complex tumour microenvironment which governs the molecular mechanisms involved in the melanoma progression is opening new therapeutic targets in these patients, especially from the perspective of FAO inhibitors and/or molecules use to prevent the release of EVs in the “tumour niche”.

## 6. Microbiota in Malignant Melanoma

During the last decade, the gut and oral cavity microbiota came to represent a key factor of tumour development by its immunomodulatory function [21,265]. It has been demonstrated that gut microorganisms are about 3.8 × 1013 with a weight of about 1.8 kg, establishing an equilibrium with the host organism, or eubiosis [266,267,268].

Moreover, recent data have demonstrated that gut microbiota, added to intratumour bacteria, may modulate the response to immunotherapy in many cancers, including melanoma, and modulate its toxic effects [268,269].

The growth of beneficial bacteria is enhanced by fibres or non-digestible compounds, or prebiotics, while healthy microbial species or probiotics are represented by *Lactobacilli*, *Bifidobacteria*, *Saccharomyces* yeasts, along with *Enterococcus*, *Bacillus*, and *Streptococcus* [270,271]. The probiotics associated to prebiotics which selectively stimulate the growth of probiotics, or the synbiotics, lead to a synergistic effect, while the use of nonviable microbial metabolites, or postbiotics, such as acetate, propionate, and butyrate (short-chain fatty acids) may mimic the effects of probiotics [272].

According to experimental studies, *Staphylococcus aureus* is stimulating Foxp3^+^Tregs, while *Enterococcus*, *Alistipes shaii*, and *Lactobacilus* are involved in Th17 and Th1 differentiation and in cytokines production [21,273]. The unbalanced microbiota, or dysbiosis, induced by antibiotics and immune checkpoint inhibitors, such as anti-CTLA-4 antibody, may led to the development of an immune-compromised tumour microenvironment [21], while *Bacteroides fragilis*, *Bacteroides thetaiotaomicron*, and *Bifidobacterium* improve the response to immunotherapy in mice models [21,274,275]. It has been also demonstrated that anti-PD-1 efficiency is higher in patients who had a microbiota rich in *Enterococcus faecium*, *Bifidobacterium longum*, *Collisella aerofaciens*, and *Ruminococcaceae* [276,277]. Anti-CTLA-4 immunotherapy results in a dominance of selected *Bacteroides* species [275]. The comprehensive analysis of commensal microbiota is valuable for detecting novel biomarkers or therapeutic targets in tumour patients treated with immune checkpoint inhibitors [21]. The abundance of gut *Ruminococcaceae bacteria*, along with *Akkermansia muciniphila* administration, contributes to the clinical response to anti-PD-1 treatment in melanoma patients [21,22]. Additionally, it has been demonstrated that patients containing *Bacteroidaceae*, *Barnesiellaceae*, and *Rikenellaceae* in their microbiota do not show colitis induced by anti-CTLA-4 therapy [278].

Furthermore, microbiota may influence immunotherapy response and toxicity, as demonstrated by the intratumour administration of CpG oligodeoxynucleotides, which mimic bacterial DNA administrated in melanoma experimental mice models, in association with an antibody against IL-10 receptor, which increase TNF production and CD8 T cells stimulation, resulting in tumour growth inhibition [273].

Based on mice experiments observations [274], faecal microbiota transfer (FMT), or the transfer of a donor entire microbial ecosystem are currently tested in clinical trials, in the immunotherapy context [274]. This is recommended mainly to patients who are refractory to anti-PD-1 treatment, with a possible pretreatment antibiotic ablation of their own microbiota [279]. Further studies would be necessary to evaluate the clinical utility of salivary or faecal microbiomes in patients which have an anti-PD-1 therapy [21].

Additional information regarding skin microbiota is currently providing correlations with UV radiations. For instance, *Malassezia furfur*, yeast which synthesizes pityriacitrin with a photoprotective role, is inhibited by UV radiations [280]. Skin colonization with *Staphylococcus epidermidis*, which produces 6-N-hydroxyaminopurine, has a preventive effect in an experimental model of photocarcinogenesis [281]. In contrast, *Trueperella* and *Fusobacterium* colonization has been associated with melanoma development in animal models [282].

These data are adding new evidence of the so-called melanoma’s “exposome”, comprised of environmental exposure, added to microbiome and genome [283].

## 7. Current Melanoma Therapeutic Approaches

### 7.1. Therapy Targets and Potential Therapeutic Biomarkers

The current therapeutic approach to melanoma depends on its location, stage, its genetic biology, or other factors. Thus, in addition to surgical therapy, immunotherapy and targeted therapy represent important recommendations as adjuvant medical therapy for advanced stages, as well as chemotherapy and radiation therapy.

The remarkable effort to decipher the genetic mutations and molecular mechanisms that underline the malignant melanomas development are useful for oncologists in choosing targeted therapies.

Numerous studies have assessed the therapeutic efficiency of c-KIT inhibitors, but the results have limited value, as most patients have eventually manifested tumour progression, possibly due to numerous cases of melanoma with central nervous system metastases, characterized by a limited drug penetration capacity [58]. Thus, current strategies are exploring combination therapy, such as an association of c-KIT inhibitors with those targeting its downstream pathways or with the immunological checkpoint blockade [65].

The concept that frequent ARID2 mutations are influencing the immune checkpoint inhibitors, being correlated to increased CD8+ T cells, supports the role of ARID2 as a potential biomarker of therapeutic efficiency of immune checkpoint inhibitors in patients with melanoma [68].

Moreover, SETD2 loss provides a potential role of this marker in melanoma therapy assessment [69].

Supplementing the surgery, radiotherapy, and immunotherapies (systemic therapy), targeted therapy is efficient, such as anti-BRAF (vemurafenib, dabrafenib, and encorafenib) and anti-c-kit (imatinib, nilotinib, and regorafenib), while anti-NRAS therapy (lonafarnib and tipifarnib) has failed in clinical trials due to NRAS activation via alternative post-translational alterations [23,70].

Targeting MAPK proteins and their regulatory components may contribute to inhibit melanoma cell genesis, thereby using them as potential therapeutic tools. Tipifarnib is targeting RAS, while Sorafenib is specifically targeting RAF. Pharmacological agents used in clinical trials, such as AZD6244, U0126, PD0325901, CI-1040, XL518, AZD8330, ARRY-162, and ARRY-300 are selective inhibitors of MEK [284], while AZ628 inhibits this pathway at the ERK level. The addition of an anti- RAS, RAF, MEK, ERK agent, MEK inhibition (MEKi) has shown considerable effects and ability to inhibit growth and induce melanoma cell death, especially in the BRAF-mutant metastatic melanoma [192]. Thus, all these pharmacological agents remain promising therapeutic targets in the MAPK pathway.

### 7.2. Therapeutic Strategies Associated to Melanoma Immune Microenvironment

A variable and complex network of interactions is characteristic for the melanoma microenvironment. The specific cells of the tumour milieu contribute to the plasticity and heterogeneity of melanoma, as they represent factors which are influencing the tumour immune escape and therapy resistance [59]. The different types of tumour cells, such as immune cells, CAFs, adipocytes, and keratinocytes, can communicate with each other or with extracellular matrix molecules, thus getting involved in the immune escape and affecting the immunosuppressive environment of the melanoma and, thus, the treatment efficiency [59]. The interaction between cancer cells and surrounding elements creates new therapeutic protocol opportunities [186]. In this direction, a therapy based on antibodies directed against adrenomedullin or an antagonist of its receptor has proven its efficiency both in vitro and in vivo [59,150].

It was revealed that CAFs and TAMs may contribute to therapy tolerance through a cytokine-signalling network that includes fibroblast-derived CXCR2 ligands and macrophage-derived IL-1β, inflammatory niches’ signalling being amplified by MAPK inhibitors, providing early drug tolerance toward BRAF and MEK inhibitors during treatment [285].

Furthermore, melanoma aggressiveness may be correlated to peritumoural mast cells density and their population may be targeted in new therapeutic approaches [286].

The modern melanoma therapy directed against immune cells is targeted against PD-1 ad CTLA-4. The best-known anti-PD-1 antibodies are nivolumab and pembrolizumab, used in the therapy of metastatic melanoma and advanced melanoma treatment, respectively. Nivolumab has shown superior therapeutic effects, improving the median progression-free survival, compared to ipilimumab, which blocks CTLA-4 inhibitory signalling pathway [59].

Considering that BRAF and MEK inhibitors can lead to changes in TME immunogenicity, therapeutic strategies consist of a combination between anti-MEK/BRAF drugs and immune checkpoint inhibitors [59]. This approach is based on the observation that BRAF-mutated melanoma cells have a low T cell infiltration and a high level of IL-6, IL-10, and VEGF, which increase the number of Tregs or myeloid-derived suppressor cells within TME [59]. Clinical investigations which have shown promising results involved the following combinations: trametinib (MEKi), dabrafenib (BRAFi), and murine anti-PD-1 antibody or cobimetinib (MEKi), vemurafenib (BRAFi), and atezolizumab (anti-PD-L1 antibody) [287,288]. Moreover, the melanoma survival was successfully improved by using combination targeted therapies for checkpoint inhibitors and MEK-ERK pathway [192].

Other immune checkpoint proteins have been studied as potential markers for new therapy strategies, such as lymphocyte activation gene-3 (LAG-3), T-cell immunoglobulin-mucin domain-containing molecule 3 (TIM-3), and T cell immunoreceptor with Ig and immunoreceptor tyrosine-based inhibitory motif (ITIM) domains (TIGIT), all of them being valuable targets for immunotherapy [59,289].

Glucocorticoid-induced tumour-necrosis-factor-receptor-related protein (GITR) represents another promising molecule for immune checkpoint therapy. GITR alteration has been shown to induce inhibition of T cell-mediated cancer cell apoptosis [290,291]. Moreover, GITR is correlated with the activity of E3 ubiquitin protein ligase neural precursor cell expressed developmentally down-regulated protein 4 (NEDD4), often involved in metastatic melanoma [290,291]. In this regard, the anti-melanoma immune response could be increased by inhibiting the activity of this enzyme [290,291].

It was demonstrated that human and mouse melanoma cells metastasis was inhibited in vitro by thymoquinone, a bioactive phytocompound, by decreasing the NLRP3 inflammasome expression, accompanied by a reduction in caspase-1 proteolytic cleavage, which led to IL-1β and IL-18 inhibition, as well as NF-κB activity suppression [116]. Moreover, the incomplete inactivation of NLRP3 inflammasome was due to reactive oxygen species (ROS) inhibition by thymoquinone. These results promote thymoquinone as a potential agent for both adjuvant immunotherapy and metastatic melanoma prevention [116].

Brain metastases of cutaneous melanoma, including acral type, react to both targeted and immune therapies, responding with targeted therapy resistance mediated by extrinsic factors. The mechanism involved is phospho-inositide 3-kinase (PI3K) pathway activation, which is a main target for brain metastases in melanoma [192].

Immune therapy’s escape of the succeeding checkpoint blockade was related to specific TME changes, represented by a lower infiltration of effector T cells, a higher number of alternatively activated macrophages, and a characteristic gene expression profile [192]. Numerous therapeutic strategies in melanoma have successfully targeted different tumourigenic mechanisms and pathways, taking into account the inflammatory microenvironment variability, such as the involvement of dendritic cells, as APCs and cytotoxic T-cell stimulators via CCR7 expression, T cell infiltration, alteration of immunosuppressive signalling pathways, neoantigen specific T cell response obtained by vaccination (mutant epitopes, mRNA, and dendritic cells) [292,293,294], antitumour immune response with oncolytic viruses [295], and antitumour activity of cytokines (IL-2 pathway and granulocyte-macrophage colony-stimulating factor—GM-CSF)) [296,297,298].

Due to heterogeneity of the melanoma immune microenvironment and weak therapeutic response to different monotherapies, attempts have been made in recent years to combine therapeutic variants in order to improve the therapeutic response, mainly with immune checkpoint inhibitors [299,300,301,302]. The promising results of combination therapy in melanoma could address the complexity of the inflammatory microenvironment and tumour heterogeneity by aiming for a therapeutic precision that exceeds the immune resistance or side effects encountered in current therapies [303].

## 8. Conclusions

Malignant melanoma exhibits a large spectrum of locations and clinicopathological characteristics, and its features are important in practical activity for diagnosis, differentials, and therapy.

Although their value is still debated, sometimes leading to controversial literature data, melanoma biomarkers may be divided into different categories, such as diagnostic, prognostic or predictive markers, progenitor and/or stem cell markers, while circulating melanoma cells or melanoma-associated extracellular molecules provide potential serum biomarkers. Biomarkers’ assessment by histological and immunohistochemical analyses of biopsies, added to pathological diagnosis and prognosis parameters, plays a very important role in melanoma management.

The melanoma microenvironment, consisting of a cell complex which comprises a population of resident or infiltrating immune cells, inflammatory mediators, adipose cells, and adipocytes-derived factors supports tumour cell proliferation, drug resistance, and metastasis. Thus, an insight into its distinct features and their interplay may reveal new pathways and molecules which prove useful in an innovative therapeutic approach.

Recent progress in immunomodulatory therapy and in manipulation of melanoma cells–adipocytes interactions has been added to the current arsenal against melanoma, and considerable efforts are made to identify suitable biomarkers for early diagnosis, staging, differential diagnosis, prognosis, and tailored therapy.

The gut and oral cavity microbiota has been recently considered a key factor of tumour development by its immunomodulatory function, and current research is aimed at modifying patients’ microbiota as an adjuvant therapy in melanoma.

Considering the complexity of the interplay between melanoma cells and their microenvironment, along with tumour heterogeneity, combination therapy is now being developed in an attempt to overcome the immune resistance and the side effects of current therapies, opening new perspectives for a better management and an improved prognosis for patients.

## Figures and Tables

**Table 1:** Malignant melanoma classification according to sun exposure, general features, and types of gene mutations.

MM Associated with UV Exposure					MM Not Consistently Associated with UV Exposure
Low-CDS (intermittent sun exposure)		High-CSD (chronic sun exposure)			mucosal melanoma; melanoma arising in congenital nevi; melanoma arising in blue nevi; Spitz melanoma; acral melanoma; uveal melanoma; nevoid melanoma; nodular melanoma
superficial spreading melanoma	subset of nodular melanoma	lentigo maligna melanoma	desmoplastic melanoma	subset of nodular melanoma	
RGP; young age; precursor lesion (nevi)	VGP; young age; precursor lesion (nevi)	RGP; old age; precursor lesion (MM in situ)	VGP; old age; precursor lesion (nevi); de novo	VGP; young age; precursor lesion (nevi)	VGP and RGP; all ages; precursor lesion (nevi)
GENOMIC					
*BRAF* (>50%); *NRAS* (25%); other mutations (rare)		*NF1*; *NRAS*; *KIT*	*NF1*; *NFKBIE*; *MAPK*	*NRAS*; *KIT*; *BRAF*; *CDKN2A*; *TP53*; *TERT*	*HRAS*; *ROS1*; *NTRK1* and *NTRK3*; *CCND1*; *TERT*; *GNAQ*; *BRAF* (10%); *KIT* (20%); *NRAS* (15–20%)

*BRAF*—B-Raf proto-oncogene, serine/threonine kinase; *CCND1*— cyclin D1; *CDKN2A*—cyclin-dependent kinase in-hibitor 2A; CSD—cumulative sun damage; *GNAQ*—G protein subunit α Q; HRAS—HRas Proto-Oncogene, GTPase; KIT—receptor tyrosine kinase; *MAPK*—mitogen-activated protein kinase; MM—malignant melanoma; NF1—neurofibromin 1; *NRAS*—neuroblastoma RAS viral oncogene homolog; *NTRK1/3*—neurotrophic receptor tyrosine kinase 1/3; RGB—radial growth pattern; *ROS1*—proto-oncogene tyrosine-protein kinase; VGP—vertical growth pattern; *TERT*—telomerase reverse transcriptase (TERT) promoter region; *TP53*—tumour protein 53; UV—ultraviolet light.

**Table 2:** Immunohistochemical markers useful in malignant melanoma diagnosis.

Marker/Protein	Locations	IHC Features	Functions
Melan A (MART1)	normal skin retina pigmented epithelium melanocytes most melanomas	high sensitivity and specificity for primary and secondary melanoma positive immunoexpression in clear cell sarcomas, PEComas or angiomyolipomas	melanocyte differentiation antigen Pmel expression, processing, traffic, and stability
MITF	dynamic subcellular location associated with variable growth and differentiation cell programs	high sensitivity in differentiation of melanoma from nonmelanocytic tumours controversial specificity in spindle cells melanoma positive immunoexpression in neurothekeoma cells, histiocytes, and mast cells	member of MiT family melanocyte development and differentiation Melan-A, Pmel, and tyrosinase transcription
HMB45	recognizes the *Silver* locus product Pmel17 located in pre-melanosomal vesicles	positive immunoexpression for melanoma and junctional nevus cells low sensitivity for metastatic melanoma positive immunoexpression in clear cell sarcomas, PEComas or angiomyolipomas	Eumelanin polymerization
SOX10	nuclei of melanocytes nuclei of breast myoepithelial cells	positive immunoexpression in melanoma, nevi, and focal positivity in desmoplastic melanoma differentiates melanoma in situ from actinic keratosis with melanocytic hyperplasia (along with MITF1) positive immunostaining of salivary and sweat glands, adenoid cystic carcinoma, atypical fibroxanthoma, granular cell tumour, and dermatofibrosarcoma protuberans	member of a family of 24 proteins involved in inflammation, cell transcription, differentiation, growth, cell cycle regulation, and calcium homeostasis transcription factor specification of the neural crest derivatives melanocytes and Schwann cells maintenance
S-100	melanocytes Langerhans cells chondrocytes glial cells Schwann cells	high sensitivity and low specificity for melanoma	inflammation cell transcription differentiation growth cell cycle regulation calcium homeostasis

HMB45—Human Melanoma Black; IHC—immunohistochemistry; MART1—melanoma antigen recognized by T cells 1; MelanA—protein melan-A; MiT—microphthalmia transcription factor; MITF—microphtalmia-associated transcription factor; PEComa—perivascular epithelioid cell tumours; Pmel—premelanosomal protein; Pmel17—premelanosomal protein17; S-100—protein S100; SOX10—SRY-related HMG-box 10 protein.

**Table 3:** Main genomic alterations in malignant melanoma.

Genes	Incidence	Pathway	Actions	MMs Type
*BRAF* (*BRAF^V600E^* *BRAF^V600^* *BRAF^V600K^* *BRAF^K601E^*)	45%	MAPK/ERK signalling	melanocytes cellular cycle, differentiation, and apoptosis	sun exposed cutaneous melanoma
	<10%			mucosal melanoma
*RAS*	15–30%	activation of downstream signalling	melanocytes proliferation, differentiation, and survival	sun-exposed cutaneous melanoma
*NRAS*	15%	MAPK/PI3K signalling	melanocytes proliferation, differentiation, and survival	sun-exposed cutaneous melanoma
*c-KIT (CD117)*	<3%	activation of MAPK and PI3K/AKT pathways	melanocytes proliferation and survival regulation	sun-exposed cutaneous melanoma
	40%			mucosal melanoma
*ATRX*	9.11%	regulation of chromosomal segregation in mitosis	melanoma progression	mucosal and cutaneous melanoma
*ARID2*	13.32%	gene transcription mechanism promoter	tumour immunity regulation	mucosal melanoma
*SETD2*	9.48%	methylation of histone H3 lysine 36	aberrant differentiation or proliferation of melanocytes	mucosal melanoma
*GNAQ/GNA11*	80–90%	MAPK signalling	melanocytes proliferation	uveal melanoma rare in cutaneous melanoma
*BAP1*	6.13%	ubiquitin-proteasome system and DNA damage response	melanocytes growth and proliferation regulation	spitzoid tumour uveal melanoma
*SF3B1*	33%	RNA splicing	tumourigenesis	mucosal melanoma
*NF1*	10–15%	downregulation of Rat Sarcoma (RAS) proteins and MAPK/PI3K signalling	melanocytes growing and survival	sun-exposed cutaneous melanomas
*RAC1*	9.2%	activated MAPK signalling	increased melanocytes proliferation and altered cell migration	sun-exposed cutaneous melanoma
*TERT*	14%	chromosomal telomere length maintenance	melanocytes survival support	cutaneous melanoma
*KRAS*	2.9%	GTPase activity	melanocytes proliferation and survival	cutaneous melanomas
*ERBB2/4*	3.29%	tyrosine kinases signalling	melanocytes proliferation and survival	cutaneous melanomas
*CDKN2A*	25–35%	RB pathway	apoptosis and melanocytes survival	cutaneous melanomas
*TP53*	15%	tumour suppressor and transcriptional activator/repressor of several downstream genes	increased melanocytes proliferation and reduced apoptosis	sun-exposed cutaneous melanomas
*PTEN*	14%	PI3K signalling	increased mitogen signalling and cell survival	cutaneous melanoma
*MAP2K1/2*	10%	MAPK signalling	melanocytes proliferation	cutaneous melanoma

*ARID2*—AT rich interactive domain 2; *ATRX*—alpha thalassemia/mental retardation syndrome X-linked; *BAP1*—BRCA1 associated protein 1; *BRAF*—B-Raf proto-oncogene, serine/threonine kinase; CD117—cluster of differentiation 117; *CDKN2A*—cyclin-dependent kinase inhibitor 2A; c-kit—mast/stem cell growth factor receptor kit; DNA—deoxyribonucleic acid; *ERBB2/4*—Erb-b2 receptor tyrosine kinase 2/4; *GNAQ*—G protein subunit α Q; *GNA11*—G-protein subunit α11; GTPase—nucleotide guanosine triphosphatase; *KRAS*—Kirsten rat sarcoma viral oncogene homolog; *MAP2K1/2*—mitogen-activated protein kinase kinase 1 and 2; MAPK/ERK—mitogen-activated protein kinase/extracellular signal-regulated kinase; MM—malignant melanoma; *NF1*—neurofibromin 1; *NRAS*—neuroblastoma RAS viral oncogene homolog; PI3K—phosphoinositide 3-kinases; *PTEN*—phosphatase and tensin homolog; *RAC1*—Ras-related C3 botulinum toxin substrate 1; *RAS*—rat sarcoma virus; *RB*—retinoblastoma tumour suppressor; *SETD2*—SET domain containing 2, histone lysine methyltransferase; *SF3B1*—splicing factor 3b subunit 1; *TERT*—telomerase reverse transcriptase (TERT) promoter region; *TP53*—tumour protein 53.

**Table 4:** Putative malignant melanoma biomarkers.

Type of Molecules	Marker
Enzymes	Cox-2 LDH Tyrosinase MMPs TIMP-1 Cathepsin K CD10 IDO Legumain
Soluble proteins and/or antigens	VEGF VEGFR-3 CRP Galectin-3 Osteopontin Heparin- and chitin-binding lectin YKL-40 MIA sICAM-1 sVCAM-1 CEACAM CYT-MAA MART-1 MAGE TA90 S100 proteins SOX
Melanin-related metabolites	L-DOPA/L-tyrosine 6H5MI2C 5-S-cysteinyl-DOPA
Circulating cell-free nucleic acids	miRNA-29c miRNA-221

6H5MI2C—6-hydroxy-5-methoxyindole-2-carboxylic acid; CEACAM—carcinoembryonic antigen-related cell adhesion molecule 1; Cox-2—cyclooxygenase-2; CRP—C-reactive protein; CYT-MAA—cytoplasmic melanoma-associated antigen; IDO—indoleamine-2,3-dioxygenase; LDH—lactate dehydrogenase; L-DOPA—L-3,4-dihydroxyphenylalanine; MAGE—melanoma-associated antigen-1; MIA—melanoma inhibitory activity; miRNA—microRNA; MMPs—matrix metalloproteinases; sICAM-1—soluble intercellular adhesion molecule 1; sVCAM-1—soluble vascular cell adhesion molecule 1; TA90—tumour-associated antigen 90; TIMP-1- tissue inhibitor of metalloproteinase-1; VEGF—vascular endothelial growth factor; VEGFR-3—vascular endothelial growth factor receptor 3.

**Table 5:** Melanoma CSCs markers.

Marker/Pathway	Function/Advantage
Nodal embryonic signalling	embryonic morphogen pluripotency maintenance enhanced tumourigenicity and metastatic ability melanoma plasticity activation due to absence of its inhibitor, Lefty putative prognostic marker
Nestin	intermediate filament associated with advanced stage
hTERT	telomerase activation by its transcriptional regulation
SOX2	regulators of CSC fate nuclear transcription factors involved in neural crest cells differentiation into melanocytes detect positive sentinel lymph nodes
SOX10	neural crest stem cell transcription factor regulation of SOX10-MITF pathway tumour cell survival, proliferation, and metastasis
CD20 (MS4A1)	self-renewal highly enrich in melanospheres tumourigenesis
CD44	EMT tumour invasion tumour metastasis
CD49d/CD29 (α4β1 integrin heterodimer)	transmembrane proteins promoter of cell proliferation and migration tumourigenesis
CD49f (Integrin α6)	
CD54/ICAM-1	cell–cell interaction
CD57/HNK-1	tumour cells adhesion, migration, and invasion
CD86/B7-2	downregulation of immune response
CD117 (c-KIT)	growth and survival of tumour cells
CD133 (prominin-1)	p38 MAPK pathway activation long-term tumourigenic potential chemoresistance metastasis induction angiogenesis
CD144 (vascular-endothelial VE-cadherin)	cell surface glycoprotein which binds to hyaluronic acid promoter of EGFR-mediated pathways tumour initiation and metastasis chemotherapy resistance
CD146/MCAM	signalling receptor tumour progression
CD166/ALCAM	tumour cell invasion and metastasis
CD271/NGFR	long-term tumour growth metastasis tumour heterogeneity
N-cadherin	potentiator of tumour cells invasiveness
miR-10b miR-21 miR200c miR-373 miR-520c	miRNA target different signalling pathways
MDR1	co-expressed with ABC transporters ABCB5 and ABCC2 self-renewal stimulation ability to form melanospheres
L1CAM	metastasis
ALDH1A1/A2/A3	self-renewal high tumourigenesis differentiation chemoresistance
ABC transporters (ABCB5, ABCG2/BCRP, ABCC1/MRP, ABCC2, ABCC6)	regulation of transport and drug exclusion from tumour cells development of multidrug resistance tumour initiation
PD-1 PDL-1	evasion of tumour immunity high tumourigenesis
CTLA-4	induces tumour proliferation suppressor of tumour cell apoptosis tumourigenesis
RANK	inducer of tumour growth and metastasis
HIF-1	promoter of tumour cells self-renewal regulator of tumour microenvironment
Snail	transcription factor
Notch4	specific signalling
γ-secretase	Notch signalling activation
Bcl-2	anti-apoptotic
GLI (Hh/Glimo)	SOX2 regulation
DDX3X	translation reprogramming metastasis
VEGFR-1	co-expression with ABCB5 vasculogenic tumour growth
VEGFR-2 VEGF Ang1/2 Tie2	angiogenic
CXCR6	high tumourigenic self-renewal
JARID1B	self-renewal high proliferative progeny tumour growth metastasis Jagged1/Notch1 signalling regulation
EZH2	epigenetic modifier promoter of tumour progression
Histone marks H3K4me2 H3K27me3 H3K9ac	epigenetic modifier gene activation/inactivation tumour progression
Hn (heterogeneous ribonucleoproteins) hnRNPs hnRNP A2B1 hnRNP I hnRNP L	splicing repressors by inhibiting splicing sites via binding to intronic or exonic site tumour progression promote the expression of anti-apoptosis genes DOCK2, TPPP3, and EIF3H

ABC transporters—ATP-binding cassette transporters; ABCB5—ABC sub-family B member 5; ABCC1 ABC sub-family C member 1; ABCC2—ABC sub-family C member 2; ABCC6—ABC sub-family C member 6; ABCG2—ABC sub-family G member 2; ALCAM—activated leukocyte cell adhesion molecule; ALDH—aldehyde dehydrogenase; Ang1/2—angiopoietin 1/2; Bcl-2—B-cell lymphoma 2; BCRP—breast cancer resistant protein; CD—cluster of differentiation; c-KIT—mast/stem cell growth factor receptor kit; CSC—cancer stem cell; CTLA-4—cytotoxic T lymphocyte antigen-4; CXCR6—C-X-C Motif Chemokine Receptor 6; DDX3X—DEAD-Box Helicase 3 X-Linked; DOCK 2—dedicator of cytokinesis 2; EGFR—epidermal growth factor receptor; EIF3H—eukaryotic translation initiation factor 3 subunit H; EMT—epithelial-mesenchymal transition; EZH2—enhancer of zeste homolog 2; GLI—Hh/Glimo; H3K27me2/3—trimethylates lysine 27 of histone H2/3; H3K9—histone H3 lysine 9 acetylation; HIF-1—hypoxia inducible factor 1; HNK-1—human natural killer 1; hnRNP—heterogeneous ribonucleoproteins; hTERT—human telomerase reverse transcriptase; ICAM-1—intercellular adhesion molecule 1; JARID1B—lysine-specific demethylase 5B; L1CAM—L1 adhesion molecular; MAPK—mitogen-activated protein kinase; MCAM- melanoma cell adhesion molecule; MDR1—multidrug-resistance gene product 1; miR—microRNA gene; MS4A1- membrane spanning 4-domains A1; NGFR—nerve growth factor receptor; Notch4—Notch Receptor 4; PD-1—programmed cell death protein 1; PDL-1—programmed death-ligand 1; RANK—receptor activator of NF-κB; Snail—Zinc finger protein SNAI1; SOX—SRY-Box transcription factor 2; Tie2—tyrosine-protein kinase receptor Tie-2; TPPP3—tubulin polymerization promoting protein family member 3; VEGF—vascular endothelial growth factor; VEGFR 1/2- vascular endothelial growth factor receptor 1/2.

## Data Availability

Not applicable.
